# Social Cognition in Adult ADHD: A Systematic Review

**DOI:** 10.3389/fpsyg.2022.940445

**Published:** 2022-07-11

**Authors:** Lucia Morellini, Martino Ceroni, Stefania Rossi, Giorgia Zerboni, Laura Rege-Colet, Elena Biglia, Rosalba Morese, Leonardo Sacco

**Affiliations:** ^1^Faculty of Biomedical Sciences, Università della Svizzera italiana, Lugano, Switzerland; ^2^Neuropsychology and Behavioral Neurology Research Unit, Neuropsychological and Speech Therapy Unit, Neurocenter of Southern Switzerland, Lugano, Switzerland; ^3^Faculty of Communication, Culture and Society, Università della Svizzera italiana, Lugano, Switzerland; ^4^Neuropsychological and Speech Therapy Unit, Neurocenter of Southern Switzerland, Lugano, Switzerland

**Keywords:** adults ADHD, social cognition, theory of mind, empathy, emotion recognition and processing, decision making, executive functions

## Abstract

The aim of this systematic review was to collect and align the research on social cognition impairments in adults with Attention-deficit/hyperactivity disorder (ADHD). In particular, we selected and analyzed papers on emotion recognition and processing, Theory of Mind (TOM), empathy, and other facets of social cognition as decision making. We identified 16 papers published between 2012 and 2022 which meet inclusion criteria. Papers search, selection, and extraction followed the PRISMA guidelines. In order to summarize data from papers, we used a narrative synthesis approach. Results show different evidence of impairment in social cognition domains in adults with ADHD. Our systematic review suggests the importance of promoting more research on this topic because it is essential to keep in mind that social cognition plays a central role in socialization and social relationships.

## Introduction

### Social Cognition

Generally speaking, for *social cognition*, we mean any cognitive, interpersonal process, or rather, any process that involves other people, whether at the group level or one-on-one (Frith and Blakemore, 2006). Social cognition includes a set of neural processes responsible for the individual's ability to “*make sense of other's behavior,”* the essential condition of social interaction (Frith and Frith, 2007). Humans are considered as social animals, and adopt certain behavior based on their interpretations of the action of others; constantly and implicitly, humans analyze, read and decode the multiple social signals from the people around them (Frith and Blakemore, 2006). At the base of these mechanisms, there are cognitive systems specialized in coping with different physical and social aspects of the world that allow us to adapt and survive. In particular, this specific cognitive system includes processes of recognition and interpretation of information from the social environment and any process aimed at understanding.their own and others' behaviors and modulating thought and actions about social demands (Frith, 2008). Social and communication abilities underlying social interactions are manifold It's possible to distinguish a set of abilities, that we share with animals, which appears in the first years of life, such as recognizing emotional expressions, and another set of more sophisticated abilities, that appears later (from 18 months) and that are typically human, such as imitating the intentional actions of others or attributing mental states, desires, and beliefs, to oneself and other people (Frith and Blakemore, 2006). The ability to establish social interactions goes through three main distinct processes:

(1) *Social Perception*: the ability to distinguish between objects and persons is a fundamental skill of social cognition, analysis of information like facial expressions, gestures, posture, body language, and voice, allows us to recognize the others as “living person” (Malle, 2015).
(2) *Social Understanding*: once the perceptual information is integrated, more sophisticated processes take action; those processes allow us to understand other's affective states—empathy (Decety and Jackson, 2004; Shamay-Tsoory and Lamm, 2018)—and the interpretation of other's observable behavior as a disposition or mental states—theory of mind and mentalizing (Premack and Woodruff, 1978; Singer, 2009; Frith and Frith, 2012; Moore et al., 2015). An individual owns theory of mind if he or she is able to impute mental states to himself or herself and to others. Such a system is regarded as a theory because these states are not observable in a direct way and, moreover, can be used to predict the behavior of other people (Premack and Woodruff, 1978).
(3) *Social Decision-Making*: social understanding leads to modulating and adapting the social behavior to social context (Malle, 2004).

On the base of these main processes, social cognition is a fundamental aspect of everyday life. In the last edition of the American Psychiatric Association's Diagnostic and Statistical Manual for Mental Disorders (DSM-5), it has been considered one of the six principal factors of neurocognitive functioning, impaired in different pathologies (Arioli et al., 2018). Social cognition impairment is a central concern in several neurodegenerative conditions such as behavioral variant of frontotemporal dementia (for example, see Palermo et al., 2020; Dodich et al., 2021), neuropsychiatric conditions (for example, bipolar or major depressive disorder), and also in neurodevelopmental conditions; in particular, in Autism (Happé, 1995) and Attention-deficit/hyperactivity disorder (ADHD; Kennedy and Adolphs, 2012). This systematic review focuses on the social cognition impairment in adults with ADHD, which is defined in the Diagnostic and Statistical Manual for Mental Disorders (DSM-5) as a neurodevelopmental disorder characterized by inattention, impulsivity, and hyperactivity. Studies indicate that over 5% of children and 2.5% of adults suffer from this disorder. ADHD is more common in males than females—with a ratio of 6:1 in children and 1, 6:1 in adults—(American Psychiatric Association, 2013). First symptoms appear already in childhood, particularly hyperactivity and inattention, which, however, improve with age. Other symptoms, including restlessness, disorganization, inattention, and impulsivity, persist in adults (American Psychiatric Association, 2013). Other studies also suggest that in adulthood there are persisting problems in emotion regulation and cognitive difficulties (Alderson et al., 2013; Mowinckel et al., 2015). In fact, ADHD doesn't only affect childhood, but persists into adult age, with a prevalence between 1 and 6% (American Psychiatric Association, 2013; Salvi et al., 2019). The relevance that this disorder is taking, it's also explained by the fact that in the older versions of DSM (for example, DSM IV), ADHD was listed under the category of child/adolescence disorder, but now is reported under the category of neuropsychological disorder, that can affect both childhood and adults (Zalsman and Shilton, 2016).

Generally, ADHD disorder in adults is associated with a high risk of developing personality disorders, and serious impairments in academic, health, occupational and social domains. In particular, dysfunction of the social domain is one of the most compromised aspects of ADHD (Nijmeijer et al., 2008). Social cognition, as previously reported, is characterized by multiple domains (Green et al., 2008) and ADHD seems to be associated with impairments in these domains (Uekermann et al., 2010). The most investigated domains of social cognition related to ADHD in adults are emotion recognition and processing and theory of mind (TOM). Still the results are ambiguous and there isn't a common line yet. Evidence in social cognition related to ADHD, in particular regarding adults, is very poor. But we know well how social cognition is important in everyday life to have and maintain a good and satisfactory quality of life. For these reasons, it's necessary to deepen and explore more the research topic.

Based on this assumption, we performed a systematic review of the current literature to align and understand the current state of the literature on the topic. Also, a descriptive review of the literature on the topic can provide the opportunity to clarify the main characteristics of social cognition deficit in adults with ADHD and to give us insight into improving social functioning in ADHD. Finally, it can provide the opportunity to understand research weaknesses to use them as starting points for future research.

## Methods

The present systematic was conducted according to the Preferred Reporting Items for Systematic Reviews and Meta-Analyses (PRISMA, Moher et al., 2009).

### Eligibility Criteria

The focus of this systematic review was to analyze studies on social cognition impairments in adults with ADHD.

The inclusion criteria were:

- Study participants (males and females) must be over 18 years old;
- Study participants must be formally diagnosed with ADHD according to the Diagnostic and Statistical Manual of Mental Disorders (Third Edition, Fourth Edition, or Fifth Edition) criteria;
- The study must include a pure ADHD group (studies with comorbidities with severe psychiatric disorders were excluded);
- The study must include at least one clinical cognitive measure (experimental measures are not allowed);
- ADHD data must be reported compared to a control group;
- Cognitive measures should not be used only for correlational scope or as predictors of non-cognitive outcomes;
- Participants are considered representative of the broadest samples of the real world.

## Information Sources

### Search Strategy

The search of the present study was conducted across Pubmed and Medline databases in line with Dodich et al. (2021). For the ADHD search strategy, we used the following terms: “adults ADHD” OR “Adults Attention-deficit/hyperactivity disorder.” The keywords were combined with “social cognition” using the Boolean term “AND” to produce the final results.

### Study Selection

**Figure 1:**
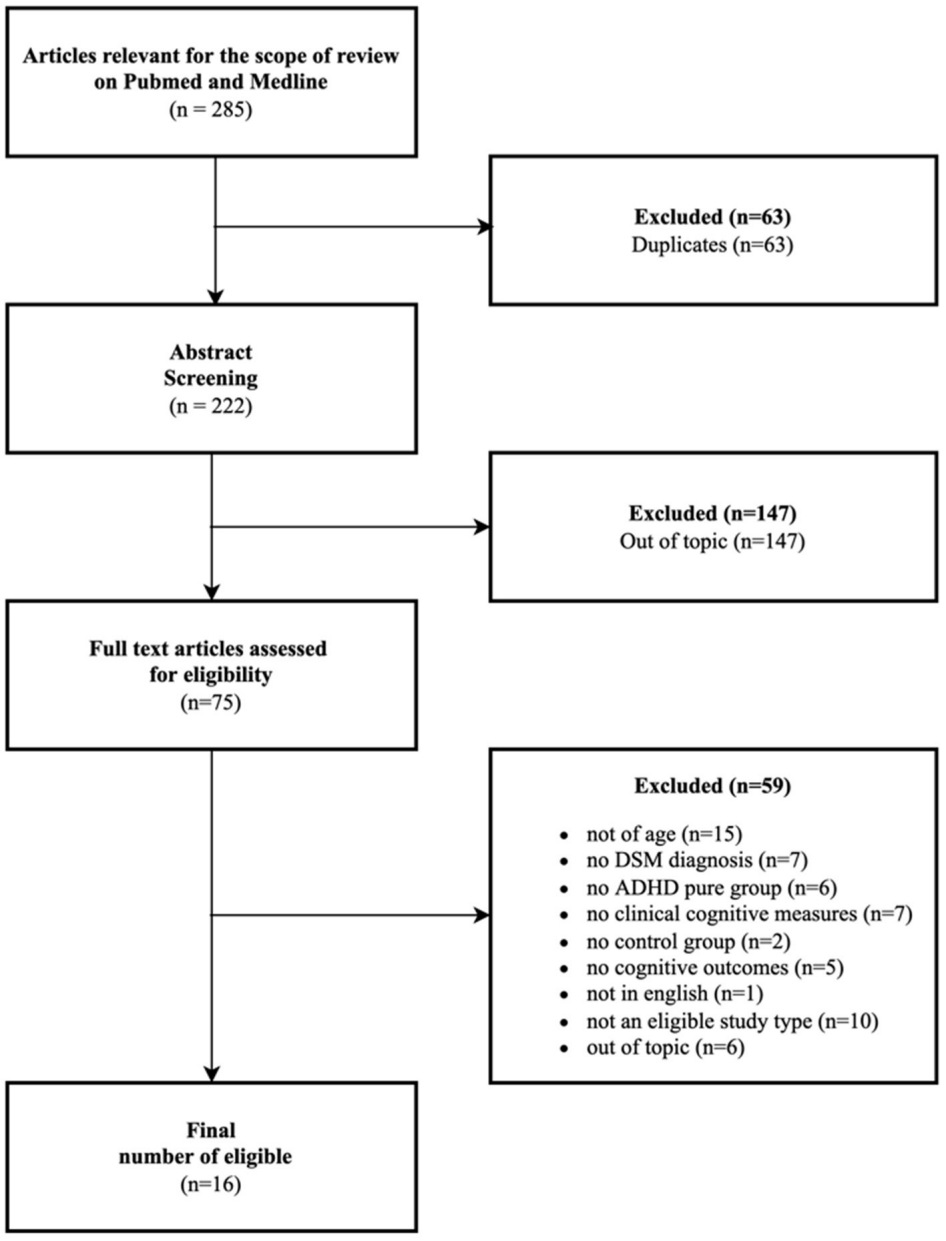
The PRISMA flow diagram.

We only considered studies limited to humans and with a limited range of periods from 2012 to March 2022. We considered the last 10 years because of the number of papers and because they are appropriate for our time and our resources available. Moreover, meta-analysis, other systematic reviews, case studies, qualitative studies, or every study with a very small sample and without quantitative measurements were excluded from the present review. Initially, the papers included in the selection were 285, and then we excluded 63 duplicates. Reading the title and abstract, from 222 articles we excluded 147 other articles from the topic. Only 75 papers were considered eligible for the scope of the present review. Those 75 papers were further analyzed by reading the complete text, to discover if they respected inclusion criteria. At this point, other 15 articles were excluded because the sample was not over 18 years old, seven articles due to the lack of diagnosis of DSM, six because the samples were not a pure group of ADHD, seven due to the lack of clinical cognitive measure, two because they did not have a control group, five because they didn't have a cognitive outcome. Other 10 papers were excluded because they were reviews or meta-analyses, others six because they were out of topic, and one because it was not in English. The final number of eligible articles for our review was 16, which were discussed in the following paragraph. Please see Figure 1 for the PRISMA flow diagram.

## Results

Overall, 16 studies were included in our review. Those studies examine the following domains of social cognition:

- Theory of mind (TOM).
- Emotion Recognition and Processing.
- Empathy.
- Decision Making.

### Theory of Mind

Six studies were included in this review (see Table 1). These studies always enrolled small groups of patients, comparing them with normal controls and, in two studies (Gonzalez-Gadea et al., 2013; Ibáñez et al., 2014), with psychiatric pathology too. The tests used for social cognition were the Reading the Mind in the Eyes Test (RMET; Baron-Cohen et al., 2001), the Faux Pas Test (FPT; Stone et al., 1998) and Movie for Assessment of Social Cognition (MASC; Dziobek et al., 2006). In four of these studies, the executive functions were examined too. The tests used to determine the deficit in executive functions were the Trail Making Test B (TMT-B; Partington and Leiter, 1949), the Stroop word color test (Stroop, 1935) and Go/No-Go from Test Battery for Attentional Performance (TAP; Zimmermann and Fimm, 2002). The RMET is a test that measures the ability to recognize social emotions and expressions. The FPT is used to assess the emotional and cognitive inference of TOM. The Movie for Assessment of Social Cognition is a video in which a man and a woman spend an evening together. The movie stops at some points. During this time, the examiner asks the subjects to answer questions regarding the social interaction of the actors. The studies reported in the tables didn't show that the Theory of Mind is compromised in the adult affected by ADHD, except for the study of Tatar and Cansiz (2022). In this study, a group of 40 adults with ADHD and 40 healthy controls were examined with the RMET, and they were significantly impaired (*p* = 0.003). In the study of Ibáñez et al. (2014), a moderate cognitive and emotional impairment of TOM was found among ADHD patients. Only one study (Mehren et al., 2021) analyzes the anatomical correlation of TOM in the ADHD population. In this group of 26 ADHD patients and 26 controls, a positive correlation was found between social cognition and the gray matter of the medial part of the superior frontal gyrus. Regarding the executive functions in Gonzalez-Gadea et al. (2013), the ADHD patients reported a higher inter-individual variability. The measures of executive functions showed a deficit in the ADHD group. In Abdel-Hamid et al., the ADHD patients showed deficits in executive functions compared to healthy controls (*p* < 0.05). These deficits were not correlated with TOM. In Tatar and Cansiz (2022), the adults affected by ADHD performed worse in TMT-B (*p* < 0.001).

**Table 1:** Overview of studies.

**Study**	**Year**	**Participants**	**Summary of relevant findings**	**Cognitive measures adopted**
**Theory of mind (*****N*** **=** **6)**				
Gonzalez-Gadea et al.	2013	22 patients with ADHD, 23 adults with Asperger's Syndrome, and 21 healthy controls.	The ADHD group showed a deficit in working memory. ADHD patients had higher inter-individual variability in executive functions.	Theory of mind (RMET and FPT), Working memory, cognitive flexibility (WCST, TMT-B), multitasking (Hotel task), decision making (Iowa Gambling Test)
Ibáñez et al.	2014	16 adults with ADHD, 14 adults with bipolar disorder, 15 schizophrenic patients, 14 first-degree relatives of patients with schizophrenia, 41 healthy controls	The early event related potential (E-N170), evoked using a face and word task, predicts the social cognitive performance. The results suggest a moderate cognitive and emotional impairment of TOM among ADHD	Theory of Mind (RME, FPT) Fluid intelligence (Raven Progressive Matrices), Processing Speed (TMT-A), Executive Functions (TMT-B)
Abdel-Hamid et al.	2019	30 adults with ADHD and 30 healthy controls	This study examines the relationship between executive functions and theory of mind, showing no influence of executive functions on theory of mind in the ADHD group examined	Theory of Mind (Movie for Assessment of Social Cognition), Executive Functions (TMT, Stroop color-word test and Go/No-Go from Test Battery for Attentional Performance)
Hayashi et al.	2020	34 adults with ADHD and 18 healthy controls	This study found a normality of implicit TOM in adults with ADHD. People with ADHD were less likely to look at the actors as a possible sign of inattention.	Video, False Belief test, Japanize version of Faux Pas Test
Mehren et al.	2021	26 adults with ADHD and 26 healthy controls	A positive correlation was found between the social cognition and the gray matter of the medial part of the superior frontal gyrus.	Theory of Mind (Movie for the Assessment of Social Cognition), Response Inhibition (go no go task), Flanker Task (Response inhibition), Verbal Intelligence (Multiple Choice Vocabulary Test)
Tatar et al.	2022	40 adults with ADHD and 40 healthy controls	Theory of Mind was impaired in adults with ADHD. Adults with ADHD performed worse on TMT part B.	Theory of Mind (RMET), Executive Functions (TMT B), Sustained Attention (Continuous performance Test)
**Emotion Recognition and Processing (*****N*** **=** **9)**				
Ibáñez et al.	2014	16 adults with ADHD, 14 adults with bipolar disorder, 15 schizophrenic patients, 14 first-degree relatives of patients with schizophrenia, 41 healthy controls.	ADHD patients have a reduced signal of N170 early event related potential for the discrimination of facial emotions. In addition, they present a deficit in processing emotional stimuli in situations with a high attentional demand.	Dual valence task (DVT): classification of words, faces or paired face–word according to their valence (positive or negative) during a two-alternative forced-choice task.
Schulz et al.	2014	14 adult males diagnosed with ADHD at 7–11 years old and 14 healthy males as controls.	The findings reveal functional abnormalities in the limbic networks of ADHD patients during a task involving cognitive control of facial emotion processing.	Face emotion go/no-go task: the correct answer depends on the valence (positive, negative or neutral) of face stimuli, corresponding to happy, sad, and neutral facial expressions.
Bisch et al.	2016	23 adults with ADHD and 31 healthy controls.	ADHD patients present deficits in social cognition regardless of other concomitant neuropsychological factors. However, they can partially compensate their deficits by a more ecological audiovisual presentation of emotional stimuli.	Classification of colored video-sequences, in which professional actors pronounce one word in neutral, happy, alluring, angry, or disgusted intonation with congruent facial expressions. Presentation *via* three different conditions: auditory, visual and audiovisual.
Kis et al.	2017	28 patients with ADHD and 29 healthy controls.	Adults with ADHD exhibit an impairment in perceiving emotional prosody, particularly for angry feelings. However, when emotional prosody is considered in relation to facial expressions, ADHD subjects do not show a significant impairment.	Tübinger Affect Battery (TAB; German version of the Florida Affect Battery): measure of the ability to perceive emotional faces and emotional prosody by watching or listening to a professional actress expressing anger, sadness, fear, joy, or emotional neutrality.
Schneidt et al.	2019	65 adults with ADHD and 49 healthy controls.	Contrary to what has been hypothesized, ADHD patients do not present an interpretation bias, such as hostile attribution bias. However, the authors evidenced a possible disturbed processing of fearful expressions in ADHD adults.	Ambivalence task: capacity to identify images presenting ambiguous emotional facial expressions (angry, happy and fearful). The ambiguity is given by the combination of different proportions of blended emotions (e.g. 70% angry and 30% happy).
Schönenberg et al.	2019	25 patients with ADHD and 25 healthy controls.	ADHD patients have a compromised recognition of sad and fearful emotional facial expressions.	Dynamic morph task: categorization of colored photographs of six different emotional facial expressions (angry, happy, fearful, sad, surprised, disgusted) presented progressively at 51 distinct intensity levels (ranging from 0 to 100%).
Thoma et al.	2020	19 patients with ADHD and 25 healthy controls.	Adults with ADHD present an impaired emotion recognition for body postures, but not for facial emotion. Furthermore, an increased amplitude of P250 ERP, in response to both emotional bodies and faces, may be related to neurocognitive processes of compensation.	Bochum Emotional Stimulus Set (BESST): classification of emotional expressions of the body and face, corresponding to basic emotions of negative (angry), positive (happy), and neutral valence.
Helfer et al.	2021	43 patients with ADHD, 14 subjects with Asperger's Syndrome and 46 healthy controls.	The correct identification of facial expressions is well-maintained in ADHD adults. However, ADHD patients are about 200 ms slower in making a correct choice. This suggests a larger speed-accuracy trade-off in facial emotion recognition.	Influential behavioral paradigm to assess facial emotion recognition (FER): a target emotion is given (anger, fear, surprise or disgust), after that all photographs showing the target emotion must be selected.
Cohen et al.	2021	19 adult males with ADHD and 16 healthy males as controls.	ADHD adults are more sensible to adverse effects of sleep deprivation on attentional functioning. This also impairs the processing of emotional facial expressions.	Visual Oddball Task: three geometric shapes (triangle, square, circle) and photographs of faces of three different male individuals with an angry or neutral expression are presented. The measure is the identification of the targets (i.e. angry faces and shapes with a cross in the center).
**Empathy (*****N*** **=** **1)**				
Kis et al.	2017	28 patients with ADHD and 29 healthy controls	The ability to empathize is a relevant deficit in ADHD patients: a lower emotional intelligence (EQ) was found in clinical population. Patients showed difficulties in perceive angry feelings.	German version of the Cambridge Behavior Scale (CBS): self-report questionnaire assessing emotional and cognitive empathy.
**Decision Making (*****N*** **=** **1)**				
Gonzalez-Gadea et al.	2013	22 patients with ADHD, 23 adults with Asperger's Syndrome and 21 healthy controls	ADHD participants perform generally worse compared to controls. However, there is no significant statistical difference (i.e., decision making skills are globally conserved in ADHD adults).	Iowa Gambling Task (IGT): 100 card selections from four decks (A & B = “high risk”; C & D = “low risk”). Each choice is rewarded or penalized by a certain number of points, depending on the degree of risk of the card. TOM: RMET, FPT. Executive functions: Backward Digit Span, Letter Number Sequencing Task from the WAIS, WCST, TMT-B, Hotel Task.

### Emotion Recognition and Processing

Of all articles included in this review, nine have content related to the recognition of emotions (see Table 1). The study samples were composed of small-medium groups of adults with ADHD (from 14 to 65 subjects), always compared to an equivalent control group of healthy people and, in two cases, also to groups containing participants with a psychiatric pathology, such as schizophrenia or bipolar disorder (Ibáñez et al., 2014) and Asperger's syndrome (Helfer et al., 2021). In each research, a different facial emotion recognition task, with also different stimuli, was used (see Table 1 for details). However, they all had in common the setting (i.e., stimuli presented in virtual format, using a computer or laptop). Concerning the measurement of the responses, all studies have considered the number or percentage of correct answers and/or the reaction time (RT). In addition, many authors have also measured intellectual performance (for example see Schneidt et al., 2019) and/or attentive-executive functioning (Ibáñez et al., 2014; Bisch et al., 2016; Kis et al., 2017; Thoma et al., 2020), in order to provide further considerations on the mechanisms underlying emotion recognition processing. Finally, in some articles, the cognitive/behavioral assessment has been coupled and completed with data from fMRI (Schulz et al., 2014), event-related potential (ERP; Ibáñez et al., 2014; Thoma et al., 2020) and Actigraph sleep recording (Cohen et al., 2021).

Schulz et al. (2014) discover functional abnormalities in the limbic networks of ADHD individuals, which negatively affect the percentage of correct answers in facial emotion recognition (*p* < 0.001). Such anomalies mainly concern the right dorsolateral prefrontal cortex (r-DLPFC), which presents a decreased connectivity with limbic structures (such as subgenual cingulate cortex, putamen, inferior frontal gyrus, and fusiform face area), compared to healthy control subjects. Additionally, during the execution of a face emotion go/no-go task, the authors have evidenced a hypoactivation of the amygdala, ventral striatum, subgenual cingulate cortex, and orbitofrontal cortex in patients with ADHD. Ibáñez et al. (2014) found a reduced signal of E-N170 ERP in ADHD adults throughout the discrimination process of facial expressions (*p* < 0.001). Additionally, they suggested that all kinds of stimuli with higher attentional demand may negatively affect the ability to process emotions in adults with ADHD. Thoma et al. (2020) highlighted no correlation between N170 ERP amplitudes and socio-cognitive abilities (*p* ≥ 0.066), but a greater amplitude of P250 ERP for neutral (*p* = 0.044) and happy (*p* = 0.011) faces, which may actually be related to a neurocognitive process of compensation of a semantic cognitive dysfunction, coupled with a reduced executive functioning (measured *via* the Letter-Number Sequencing Task from the WAIS, Stroop test and TMT-B) and alterations of emotional processing. Moreover, they stated that ADHD patients showed no difficulties in recognizing emotional facial expressions (*p* ≥ 0.300). Schönenberg et al. (2019) described a stable deficit of emotion recognition for sad (*p* = 0.029) and fearful (*p* = 0.017) facial expressions in ADHD adults. Kis et al. (2017) reported that individuals with ADHD have difficulties in perceiving statements of anger, compared to other emotions. This result was even more significant when items of anger were associated with emotional prosody of anger. Authors refer that this result might be explained by a misunderstanding between individuals and society due to an alterate perception on emotional signals, that inevitably causes problem in communication and interpretation. Bisch et al. (2016) found social cognition disturbances in ADHD patients (*p* < 0.050), of which they are fully aware, and that are independent of neuropsychological factors such as attention (measured *via* “Sustained attention” and “Alertness” sub-tests from TAP) or verbal intelligence (measured via the Mehrfach-Wortschatz Intelligence Test, MWT-B; Kis et al., 2017). However, ADHD participants manifested a great gain in emotion recognition accuracy during the audiovisual presentation of the stimuli (*p* < 0.050). Schneidt et al. (2019) highlighted that ADHD participants do not present an interpretation bias, such as hostile attribution bias (HAB). However, it seems that social information processing is globally compromised in ADHD, especially regarding the perceptual coding phase of fearful expressions, while the interpretation is not significantly impaired. Helfer et al. (2021) found no difference in the ability to recognize facial expressions of anger (*p* = 0.329), fear (*p* = 0.775), disgust (*p* = 0.670) and surprise (*p* = 0.234) between adults with ADHD and healthy controls. However, they described a slowdown of ADHD subjects in categorizing emotion, probably related to the mind wandering effect (measured *via* the Mind Excessively Wandering Scale, MEWS; Mowlem et al., 2019). Cohen et al. (2021) found that sleep deprivation affects the attentional resources of adults with ADHD more than those of healthy controls; this has an impact on the categorization of emotional expressions highlighted by an increase in reaction time (*p* < 0.001) and the number of error (*p* < 0.001).

### Empathy

Just one pilot study, with a small sample size investigating empathy in ADHD, has been selected. Kis et al. (2017) used a self-report questionnaire, the German version of the Cambridge Behavior Scale (CBS; Baron-Cohen and Wheelwright, 2004; see Table 1), to evaluate emotional intelligence (EQ). Specifically, they used this test to examine empathy. The questionnaire includes 60 items, 40 refer to empathy, and 20 refer to distraction in order to eliminate the possibility of automatism on a 4-point Likert.

Authors (Kis et al., 2017) refer that CBS can assess both emotional and cognitive empathy, but if we take a look at single items of the questionnaire, there aren't specific items for emotion or cognition, they are all generic without any distinction. This makes the test not very exhaustive in the evaluation of empathy. A questionnaire like IRI (Interpersonal Reactivity Index, Davis, 1980) would have been more comprehensive because, compared to CBS, using 28 items on 5-point Likert, gives us specific results on four different aspects of empathy: perspective taking (PT), fantasy scale (FS), empathic concern scale (EC) and personal distress. Using these four sub-scales provides us not only results on cognitive/emotional empathy but also a multidimensional approach to evaluate empathy.

The authors found a lower EQ of patients compared with EQ of the controls (*P* < 0.001), with worse performances in the tasks measuring emotional prosody (especially a difficulty in the perception of emotional angry statements), and intermodal skills. Furthermore, the ability to empathize was not related to executive functions and there were no gender differences in the patient group.

### Decision Making

Concerning decision making, only one article has been selected for this review. The study of Gonzalez-Gadea et al. (2013) compared the performance on the Iowa Gambling Task (IGT; Bechara et al., 1994) (please see Table 1 for details) of 22 subjects with ADHD, 23 adults with Asperger's Syndrome, and 21 healthy controls. Globally, the authors found no difference in this task between ADHD participants and healthy controls (*p* = 0.200), even if adults with ADHD generally perform worse in the second block of the IGT task (*p* < 0.01).

## Discussion

This systematic review aims to analyze the literature available on recent studies by dividing them into subtypes of social cognition: Theory of mind, Emotion Recognition and Processing, Empathy, and Decision Making.

### Theory of Mind

Research on social cognition has been increasing in recent years. It is a topic of great clinical interest, as it is involved in both psychiatric and neurodegenerative diseases. ADHD, with its peculiar deficits, raises important questions on this research topic. The Meta-analysis of Bora and Pantelis (2016) has given us the first answers, especially in the child and adolescent population. ADHD patients are intermediate between autism spectrum disorders and healthy subjects in the deficit of social cognition in the adolescent group. An important answer is now awaited in the adult population, where more studies are needed.

The studies reviewed on the theory of mind were only six. The patients analyzed in the single studies were small groups, between 16 (Ibáñez et al., 2014) and 40 (Tatar and Cansiz, 2022) participants. These data don't give the results a high relevance. There are still two possible interpretations: (1) Theory of mind is not compromised in adults with ADHD because of the neuronal development and maturation of the brain and personal experience in adolescence (Gonzalez-Gadea et al., 2013; Bora and Pantelis, 2016; Abdel-Hamid et al., 2019). (2) Theory of mind is compromised, and deficits are related to executive functions and cognitive flexibility (Tatar and Cansiz, 2022). Reviews that analyzed attention, inhibition, and executive functions demonstrate a deficit in these domains (Hervey et al., 2004; Boonstra et al., 2005). It is not possible to make inferences between the two subcomponents of the theory of mind. There are insufficient data to sustain a deficit in the affective or cognitive subcomponent (Kemp et al., 2012). The tests that analyzed the two subcomponents are often not presented in the same study. These tests are the “false belief” for the cognitive component and “reading the mind in the eyes” for the affective component. The “faux pas test” can analyze both components, but it is not possible to have a differentiation between the two subdomains. At the same time, there is no evidence of the relationship with the anatomical areas. The only available study did not confirm the known involvement of the dorsolateral and the ventromedial prefrontal cortex (Shamay-Tsoory et al., 2006; Kalbe et al., 2010).

### Empathy

Empathy is an important construct of emotional intelligence and understanding whether it is impaired in some clinical conditions could explain and justify several situations and it could be the basis for future treatments. Kis et al. (2017) described a lack of empathy skills in adult patients with ADHD. The authors found that the ability to perceive angry emotional statements was particularly compromised regardless of the intellectual performance, presence of executive disorders, and other underlying psychiatric diseases. Given that the study focused on empathy in ADHD adult patients fulfilling our inclusion criteria was just a pilot one (Kis et al., 2017), in future research it might be interesting to fully understand within larger sample sizes whether the ability to empathize is compromised in the clinical population. For the assessment of dispositional empathy, the Interpersonal Reactivity Index (IRI; Davis, 1983) and a behavioral task as the RMET have already been used by Thoma et al. (2020), but authors performed just correlations between ERP measures and background data, without focusing on the empathy competences between the group of patients and controls. For this reason, we didn't consider this study in our systematic review.

### Emotion Recognition

Thanks to the acquisition of fMRI images, Schulz et al. (2014) highlighted functional abnormalities in the limbic networks of ADHD individuals, which negatively affect facial emotion processing. Such anomalies concern the r-DLPFC, which presents a decreased connectivity with limbic structures, and hypoactivation of the amygdala, ventral striatum, subgenual cingulate cortex and orbitofrontal cortex. Moreover, Ibáñez et al. (2014) have found a reduced N170 ERP signal in ADHD adults during the discrimination process of facial expressions. On the contrary, a more recent study evidenced no correlation between N170 ERP and socio-cognitive abilities, but a greater amplitude of P250 ERP, which may actually be related to a neurocognitive process of compensation (Thoma et al., 2020). Also, concerning behavioral and cognitive analyzes, the results highlighted are not always totally consistent. Indeed, Schönenberg et al. (2019) describe a stable deficit of emotion recognition only for sad and fearful facial expressions in ADHD adults, while Thoma et al. (2020) state that ADHD patients showed no difficulties in recognizing emotional facial expressions, but only for body postures. In addition, Kis et al. (2017) evidenced an impairment in the processing of emotional prosody alone, while when it is presented with a consistent facial expression, the deficit is no longer significant. Moreover, according to Schneidt et al. (2019), ADHD subjects do not present a full-blown disturbance of interpretation of facial expressions (such as the HAB), even if their social information processing would anyway seem partially compromised. To explain these seemingly inconsistent results, Bisch et al. (2016) also found social cognition disturbances in ADHD patients. However, they hypothesized the presence of a mechanism of partial compensation of the difficulties in social cognition, thanks to the audiovisual presentation of the stimuli. This hypothesis is also supported by other evidence that has described the benefit of a more ecological (dynamic) presentation of facial expressions on the emotion recognition process (Bould and Morris, 2008; Krumhuber et al., 2013). In this direction, Ibáñez et al. (2014) suggest that all kinds of stimuli with higher attentional demand may negatively affect the ability to process emotions in adults with ADHD. Consistently, Helfer et al. (2021) have described a slowdown of ADHD subjects in categorizing emotion, probably related to the mind wandering effect. Furthermore, Cohen et al. (2021) found that sleep deprivation affects the attentional resources of adults with ADHD with a consequential impact on facial emotion processing.

In short, to summarize the results concerning the recognition of emotions in adults with ADHD, it would seem that these patients are globally able to compensate for the recognition of emotion itself, despite having limitations in the cognitive treatment and processing of the same mainly due to a dysfunction of the limbic network. Furthermore, these limitations seem to depend on: the kind of emotion; the mode of presentation of the emotion (ecological vs. non-ecological; complete vs. partial; facial expression vs. body posture), the attentional demand of the stimulus (high vs. low); the comorbid presence with mind wandering.

### Decision Making

One study on decision making, Gonzalez-Gadea et al. (2013), concluded that decision making abilities are globally conserved in ADHD adults, even if they generally perform worse than healthy subjects. Even though social decision making it's been described as an important aspect of social cognition (see Section INTRODUCTION, line 69) it's reported that social understanding leads to modulating and adapting the social behavior to social context (Malle, 2004), results and research on this topic are very poor. Within our review we include only one paper on decision making (Gonzalez-Gadea et al., 2013), whom it's also very lacking results and was treated in one paragraph for correlational purposes only. This marks a big limitation and gap in the completeness of our review, but at the same time, it's a starting point for future research.

## Conclusion

This systematic review analyzed the recent literature on social cognition in ADHD adults. Social cognition considers more facets; sometimes this implies more measures to assess the several processes of social cognition. This can represent a gap in the case of comparisons between studies. It's important to underline that the studies included in the review aren't evaluated in the same manner by the authors. The same concepts were investigated using different instruments, this can represent a limitation. Nevertheless, our goal was to collect studies that deal with the different domains of social cognition, so this limit hasn't influenced our work, and at the same time it could be a starting point for future research. In fact, it might be interesting for future researchers to explore all the tools used to estimate the main domains of social cognition, with the ultimate purpose of understanding, which among them, might be the most comprehensive.

Typically, social cognition is investigated mainly through neuropsychological exams, but there are also other tools that could give added value to psychological research (i.e., fMRI, PET studies, etc.). Another limitation that could be a starting point for the future is that studies that deal with empathy and social decision-making are very poor, so we suggest and encourage to deepen more those research topics.

In addition, a small size sample identified in some studies could be the main gap based on a limited number of researches on this topic. We underline the importance of increasing interest in this research topic in order to fill the main gaps in future studies.

## Data Availability Statement

The raw data supporting the conclusions of this article will be made available by the authors, without undue reservation.

## Author Contributions

LS carried out part of the literature search, collected part of the studies, described part of the results, elaborate part of the results and discussion, wrote part of the manuscript, and reviewed the final version manuscript. RM elaborated table and part of the manuscript (conclusion), reviewed the final version of the manuscript, and supervised the systematic review. LM had the main contribution to literature search and selection, created the draft, the table and the flow diagram, wrote the introduction and method, and reviewed the references and the final version of the manuscript. SR collected part of the literature search and described part of the results and discussion. MC contributed to the collection and selection of the literature, the editing of the table, the drafting of part of the results, and discussion and reviewed the final version of the manuscript (in particular with regard to references). GZ and EB contributed to the collected part of the literature search. LR-C contributed to the collection and selection of the literature. All authors contributed to the article and approved the submitted version.

## Funding

Open access funding provided by Università della Svizzera italiana.

## Conflict of Interest

The authors declare that the research was conducted in the absence of any commercial or financial relationships that could be construed as a potential conflict of interest.

## Publisher's Note

All claims expressed in this article are solely those of the authors and do not necessarily represent those of their affiliated organizations, or those of the publisher, the editors and the reviewers. Any product that may be evaluated in this article, or claim that may be made by its manufacturer, is not guaranteed or endorsed by the publisher.
