# Corrigendum: Social cognition in adult ADHD: a systematic review

**DOI:** 10.3389/fpsyg.2024.1509848

**Published:** 2024-12-10

**Authors:** Lucia Morellini, Martino Ceroni, Stefania Rossi, Giorgia Zerboni, Laura Rege-Colet, Elena Biglia, Rosalba Morese, Leonardo Sacco

**Affiliations:** ^1^Faculty of Biomedical Sciences, Università della Svizzera italiana, Lugano, Switzerland; ^2^Neuropsychology and Behavioral Neurology Research Unit, Neuropsychological and Speech Therapy Unit, Neurocenter of Southern Switzerland, Lugano, Switzerland; ^3^Faculty of Communication, Culture and Society, Università della Svizzera italiana, Lugano, Switzerland; ^4^Neuropsychological and Speech Therapy Unit, Neurocenter of Southern Switzerland, Lugano, Switzerland

**Keywords:** adults ADHD, social cognition, theory of mind, empathy, emotion recognition and processing, decision making, executive functions

In the published article, there was an error. The OSF reference was incorrectly written.

A correction has been made to **Methods**, This sentence previously stated:

“The present systematic was conducted according to the Preferred Reporting Items for Systematic Reviews and Meta-Analyses (PRISMA, Moher et al., 2009). The method is currently available in the Open Science Framework (OSF, https://osf.io/hn7mc/).”

The correct sentence appears below:

“The present systematic was conducted according to the Preferred Reporting Items for Systematic Reviews and Meta-Analyses (PRISMA, Moher et al., 2009).”

The correspondence email address for author Lucia Morellini has also been updated. Instead of ‘lucia.morellini@usi.ch’, it should be ‘lucia.morellini@eoc.ch’.

The authors apologize for this error and state that this does not change the scientific conclusions of the article in any way. The original article has been updated.

